# An all-silicon design of a high-efficiency broadband transmissive terahertz polarization convertor

**DOI:** 10.1007/s12200-023-00098-9

**Published:** 2023-12-06

**Authors:** Xiaohua Xing, Die Zou, Xin Ding, Jianquan Yao, Liang Wu

**Affiliations:** College of Precision Instrument and Optoelectronics Engineering, Key Laboratory of Optoelectronics Information and Technology (Ministry of Education), Tianjin University, Tianjin, 300072 China

**Keywords:** Broadband, High efficiency, Polarization conversion, All-silicon

## Abstract

Polarization, a fundamental behavior of electromagnetic waves, holds immense potential across diverse domains such as environmental monitoring, biomedicine, and ocean exploration. However, achieving efficient modulation of terahertz waves with wide operational bandwidth poses significant challenges. Here, we introduce an all-silicon polarization converter designed specifically to operate in the terahertz range of the electromagnetic spectrum. Simulation results demonstrate that the average conversion efficiency of cross-linear waves exceeds 80% across a wide frequency range spanning from 1.00 to 2.32 THz, with the highest conversion efficiency peaking at an impressive 99.97%. Additionally, our proposed structure facilitates linear-to-circular polarization conversion with an ellipticity of 1 at 0.85 THz. Furthermore, by rotating the cross-shaped microstructure, active control over arbitrary polarization states can be achieved. To summarize, the proposed structure offers remarkable flexibility and ease of integration, providing a reliable and practical solution for achieving broadband and efficient polarization conversion of terahertz waves.

**Graphical abstract:**

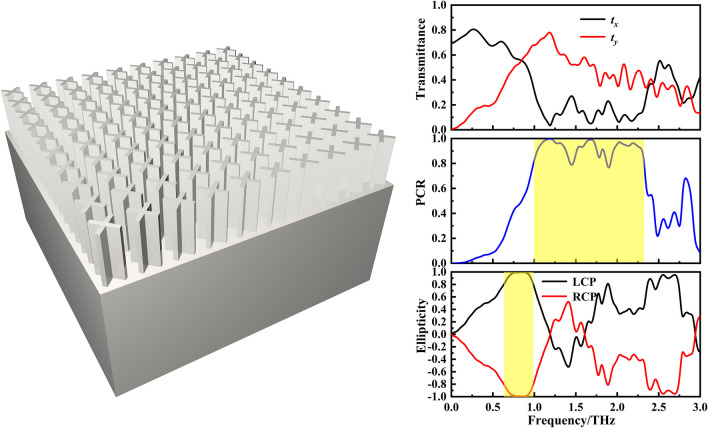

## Introduction

With the significant advancements in terahertz (THz) science and technology, terahertz waves have become indispensable in radar [1], communication [2, 3], and imaging [4, 5]. These applications demand highly efficient optical devices, including lenses, wave plates, switches, and polarization rotators. Among these devices, polarization converters are crucial for controlling polarization and are typically constructed using optical gratings [6], birefringent crystals [7, 8] or anisotropic materials. However, traditional converters suffer from drawbacks such as large size, susceptibility to damage, and integration challenges [9, 10]. Moreover, the conventional natural materials chosen for traditional converters have significant limitations in electromagnetic response. Consequently, researchers are considering the use of metamaterials as substitutes for traditional materials.

Metamaterials [11, 12], as artificial composite materials, have gained substantial attention due to their unique electromagnetic properties, offering alternative possibilities for controlling the polarization of terahertz waves [13–17]. In 2018, Sun et al. demonstrated a transmissive metal polarization rotator with a double-layer structure, which, relative to pre-existing technologies, exhibited reduced loss and improved polarization conversion performance [18]. Cheng et al. further utilized chiral metamaterials to design a versatile terahertz polarization converter, achieving linear-to-circular polarization conversion at 1.14 and 1.34 THz, as well as cross-linear polarization conversion between 2.19 and 2.47 THz [19]. In 2020, Zi et al. proposed a transmissive dual-function terahertz wave plate based on all-dielectric metamaterials, enabling two different polarization conversions at 1.01 THz [20]. These designs operate in the transmissive mode, thereby to some extent avoiding the associated drawbacks of traditional converters and facilitating easy integration. However, their effective working bandwidth remains relatively narrow. Therefore, achieving a transmissive broadband device with high polarization conversion efficiency is important for development of control of the polarization state of terahertz waves.

In this paper, we present a broadband all-silicon transmissive multi-functional polarization converter with high efficiency, employing cross-shaped microstructures. This design enhances the effective electromagnetic response, thereby improving the device efficiency. In comparison to previously reported converters with limited working bandwidth, our proposed design offers a significantly broader operational range. Numerical simulations confirm the device’s capability to achieve polarization conversion for both cross-linear and linear-to-circular polarized waves in the terahertz frequency range. The proposed device holds potential applications in terahertz spectroscopy, imaging, and communications.

## Theoretical model

**Fig. 1:**
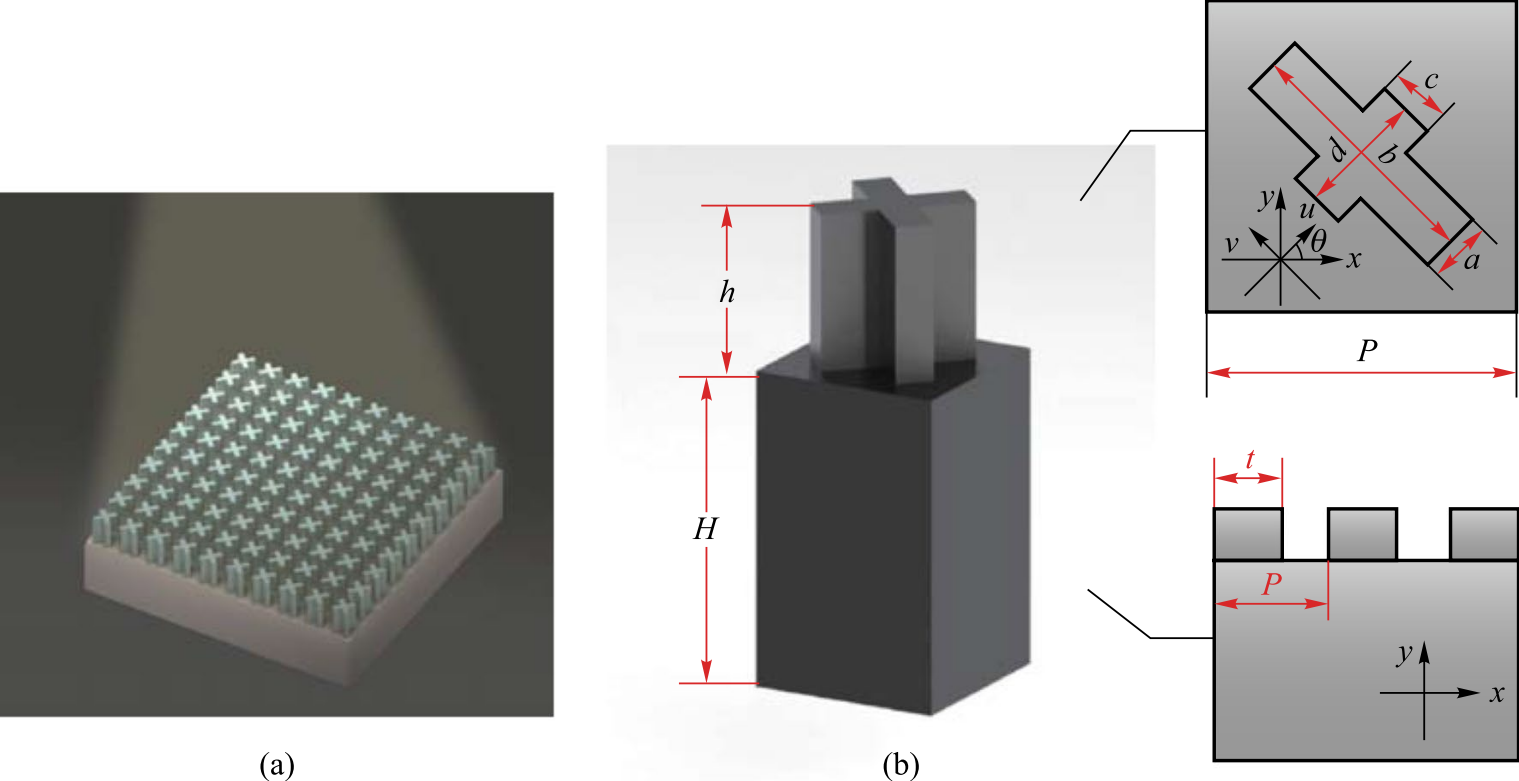
Schematic diagram of the designed cross-shaped microstructure. **a** Schematic of proposed convertor. **b** Stereograph of microstructure. Here, *h* represents height; *H* is substrate thickness; *a–d* show length and width; *P* is period; *θ* is the included angle between the cross-shaped secondary axis and the *x*-axis;* t* is the length of high-resistance silicon

The unit cell of this polarization convertor is composed of a cross-shaped patch on top of a dielectric substrate (schematic in Fig. 1b). When a beam of terahertz waves passes through this device, its polarization direction will change. Here, $E_{{\text{i}}} = E_{{{\text{i}}x}} {\text{e}}^{{ - {\text{j}}kz}}$ is the incident polarized wave amplitude, *E*_i*x*_ is incident wave amplitude, *k* is the wave number, and *E*_i*x*_ represents the amplitude of the incident wave along the *x-*axis [21]. Then the transmitted wave can be expressed

1 $$E_{{\text{t}}} = E_{{{\text{t}}x}} {\text{e}}^{{{\text{j}}\varphi_{x} }} {\text{e}}^{{{\text{j}}kz}} ,$$

where *E*_t*x*_ and *φ*_*x*_ represent the amplitude and additional phase of the transmitted wave, respectively.

If the cross-shaped microstructure in *u-v* coordination rotates through angle *θ* (*θ* is the included angle between the cross-shaped minor axis and the *x*-axis, as shown in Fig. 1b), the incident and transmitted wave amplitude in the *x*-direction can be expressed as [22]

2 $$E_{{\text{i}}} = \left( {\cos \theta - \sin \theta } \right)E_{{{\text{i}}x}} {\text{e}}^{{{\text{j}}kz}} ,$$

3 $$E_{{\text{t}}} = \left( {\cos \theta {\text{e}}^{{{\text{j}}\varphi_{u} }} - \sin \theta {\text{e}}^{{{\text{j}}\varphi_{v} }} } \right)E_{{{\text{t}}x}} {\text{e}}^{{{\text{j}}kz}} .$$

The cross-shaped microstructure does not change *θ*, so for simplicity we usually consider that: *φ*_*x*_ = *φ*_*u*_ and *φ*_*y*_ = *φ*_*v*_. The transmitted waves are then given by

4 $$E_{{\text{t}}} = \left[ {\left( {\cos \theta + \sin \theta } \right)\cos \theta {\text{e}}^{{{\text{j}}\varphi_{x} }} - \left( {\cos \theta + \sin \theta } \right)\sin \theta {\text{e}}^{{{\text{j}}\varphi_{y} }} } \right]E_{{{\text{t}}x}} {\text{e}}^{{{\text{j}}kz}} .$$

Then the polarization state of transmitted waves is related to the rotated angle and the phase difference between the two orthogonal coordinate axes. The linear polarization direction of transmitted wave will rotate π/2 as long as the two polarization components along the *x*- and *y*-axis produce a phase difference of π [23],

5 $$\varphi = \frac{{2{\uppi }}}{\lambda }\left( {n_{u} - n_{v} } \right)h = {\uppi ,}$$

where *λ* is the wavelength of incident wave, *n*_*u*_ and *n*_*v*_ are the refractive indices in *u-* and *v*-directions, respectively, and *h* is the thickness of cross-shaped microstructure. Since $n = \sqrt {\varepsilon \mu }$, the modulation of polarization state is related to permittivity [24], which is a unique and significant characteristic of metamaterials. We can handle the polarization direction through artificially changing the shape, size and distribution of the unit cell [25, 26]. It can be seen from Fig. 1b that the high-resistance silicon and air are arranged periodically. Therefore, the effective permittivity calculation formulas in the *u*-direction and *v*-direction can be expressed as

6 $$\left\{ \begin{aligned} &\varepsilon_{{{\text{eff}}}} = f\varepsilon_{1} + \left( {1 - f} \right)\varepsilon_{2} , \hfill \\ &f = \frac{t}{T}, \hfill \\ \end{aligned} \right.$$

where *ε*_1_ and *ε*_2_ are the permittivities of high-resistance silicon and air, respectively, *ε*_eff_ is the effective permittivity, and *f* is the duty cycle of high resistance silicon. By appropriately adjusting the proportion of the high-resistance silicon and changing the refractive index of the convertor, the polarization direction of terahertz waves can be deflected toward the target angle.

From Eq. (4), the transmitted waves of *x*- and *y*-polarized components are also related to the rotated angle *θ*. When Δ*φ* = *φ*_*y*_ − *φ*_*x*_ = π, the transmitted wave amplitudes are $E_{{\text{t}}} = - (\cos 2\theta + \sin 2\theta ){\text{e}}^{{{\text{i}}\varphi_{x} }} {\text{e}}^{{{\text{j}}kz}}$. Therefore, when *θ* = 45°, the *x*-polarized incident waves can be completely turned into *y*-polarized waves. According to Eq. (4), we set *θ* = 45° and the polarization direction of incident waves is then along the *x*-axis. The transmittance in *x-* and *y*-polarized directions are *t*_*x*_ = *E*_*x*_/*E*_i_, *t*_*y*_ = *E*_*y*_/*E*_i_. The polarization conversion rate (PCR) is defined as PCR = *|t*_*y*_*|*^2^/(*|t*_*x*_*|*^2^ + *|t*_*y*_|^2^). Here, *E*_*x*_ and *E*_*y*_ represent the output electrical field intensity of the terahertz waves polarized along *x*- and *y*-axis, respectively, *E*_i_ represents the electrical field intensity of incident terahertz waves.

To verify the design, the simulation is carried out in CST MWS, where the material is high-resistance silicon with *ε* = 11.9 and *μ* = 1.0. In the simulation, the periodic boundary condition in the *x–y* plane is applied to simulate the periodic array state. Meanwhile, the open condition along the *z*-direction is set to match the practical incident and transmitted terahertz wave paths. For the light source setup, we use a linearly polarized wave along the *x*-direction incident along the − *z*-axis to the metamaterial.

## Results and discussion

### Influence of parameters on device performance

There are several factors that affect the device performance: length, width, height, period, and substrate thickness. By varying these factors, we can obtain the influence of structural parameters on PCR, and the optimal structural parameters.

Table 1 shows the parameter scanning range of the cross-shaped microstructure. Through the parameter scanning, the optimal solution of PCR and the effective working bandwidth are obtained.

**Table 1:** Scanning range of cross-shaped microstructure parameters

Structural parameters (see Fig. 1)	Parameter scanning range/μm	Optimal value/μm
*a*	10–30	30
*b*	70–90	80
*c*	10–30	30
*d*	30–50	40
*P*	80–180	80
*h*	100–300	180

**Fig. 2:**
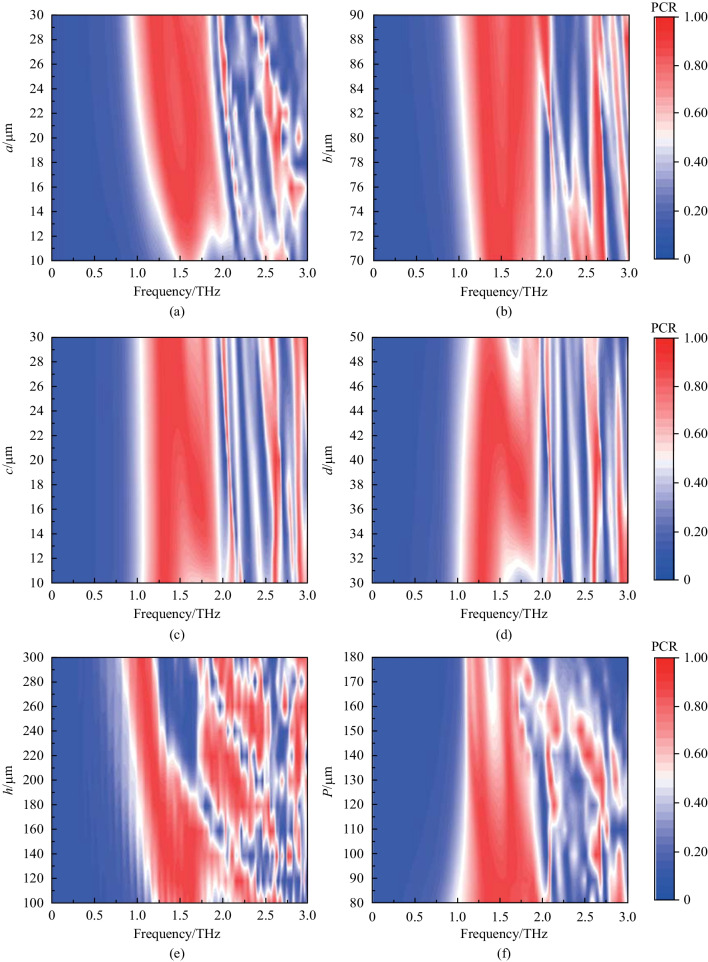
Parameter scanning results of the microstructure. Here, the initial parameters are *a* = 10 μm, *b* = 70 μm, *c* = 10 μm, *d* = 30 μm,* h* = 150 μm, *P* = 100 μm. **a**–**e** Show the influence of the cross-shaped structure parameters on PCR, respectively. **f** shows the influence of the period of the microstructure on PCR

Figure 2 illustrates the simulated PCR distributions for various structural parameters. (Here, the red area indicates that the device has high polarization conversion efficiency in this frequency band). The red areas represent PCR ≥ 80%. These area are concentrated on the range of 1.00–2.5 THz. Figure 2a–e show the impact of the cross-shaped parameters on the effective working bandwidth and PCR. When *a* = 30 μm, *c* = 30 μm, the device performance is the best. Figure 2b, d, e indicate that the effective working bandwidth increases first and then drops off, with the maximum at *b* = 80 μm,* d* = 40 μm,* h* = 180 μm. As shown in Fig. 2f, the period has great influence on device efficiency. With the period increasing, the effective working frequency range continuously reduces, and reaches the best value at *P* = 80 μm. By analyzing the influence of microstructure parameters on the polarization conversion, the optimum parameter combination of the cross-shaped microstructure is as follows: *a* = 30 μm; *b* = 80 μm; *c* = 30 μm; *d* = 40 μm; *h* = 180 μm; *P* = 80 μm.

### Function of broadband half-wave plate

**Fig. 3:**
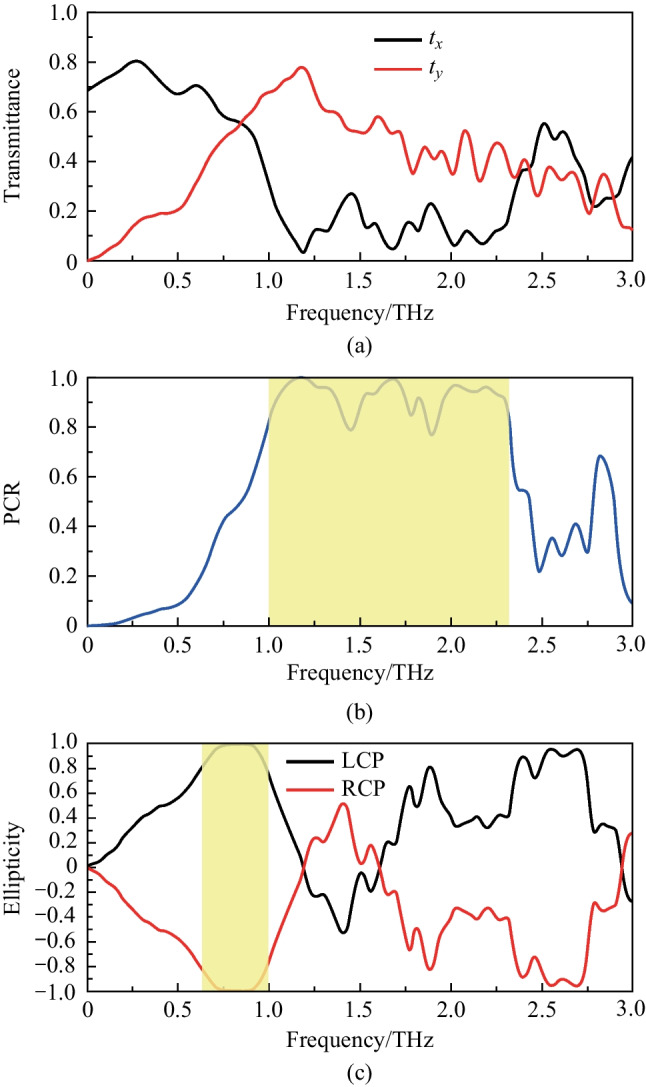
Simulation results of the polarization convertor. **a** Transmittance and **b** PCR of the cross shaped microstructure at the optimal parameters. The yellow area represents PCR exceeding 80% in this frequency range. **c** Performance of the device as a quarter wave plate at different incident polarized waves. The yellow area represents *χ* ≥ 80%. Here, *a* = 30 μm; *b* = 80 μm; *c* = 30 μm; *d* = 40 μm; *h* = 180 μm; *P* = 80 μm; *H* = 500 μm

According to the optimal structural parameters obtained by parameter scanning, the microstructure is then simulated. Figure 3 shows the transmittance and corresponding PCR of the device at the optimal parameters as determined by simulation. It can be understood that in the frequency range of 1.00–2.32 THz, PCR is greater than 80%, representing a very broad effective working bandwidth. In addition, Table 2 gives the performance of the proposed structure in comparsion with previous works. Compared with previously reported designs, our design for cross-polarization conversion has simple structure, wide operating bandwidth, and works well at wide incidence angles.

**Table 2:** Performance comparsion of the proposed struture with previous works

References	Structure configuration	Operating mode	Method	Effective bandwidth/THz
[27]	Full dielectric elliptic column	Transmissive	The polarization conversion based on spatially staggered anisotropic birefringence effect	0.4 (PCR ≥ 70%)
[28]	Three-layer metal structure	Transmissive	The polarization conversion based on wavefront manipulation	0.6 (PCR ≥ 90%)
[29]	multilayer metamaterial	Transmissive	The polarization conversion of peak resonances based on GST225 material	0.72 (PCR ≥ 70%)
[30]	Metal ring + multilayer film	Reflective	The polarization conversion based on VO_2_ phase transition properties	0.58 (PCR ≥ 90%)
[31]	Z-shaped metal antennas + rods	Transmissive	The polarization conversion based on multiple interference models	0.32 (PCR ≥ 80%)
This work	Cross-shaped pillars	Transmissive	The polarization conversion based on all dielectric metamaterial	1.32 (PCR ≥ 80%)

### Function as a quarter-wave plate

In addition, this device can be used not only as a half-wave plate, but also as a quarter-wave plate. The quarter wave plate can convert linearly polarized waves into circularly or elliptically polarized waves. The expression of the quarter-wave plate can be expressed as

8 $$\varphi = \frac{{2{\uppi }}}{\lambda }\left( {n_{u} - n_{v} } \right)h = \frac{{\uppi }}{2}.$$

**Fig. 4:**
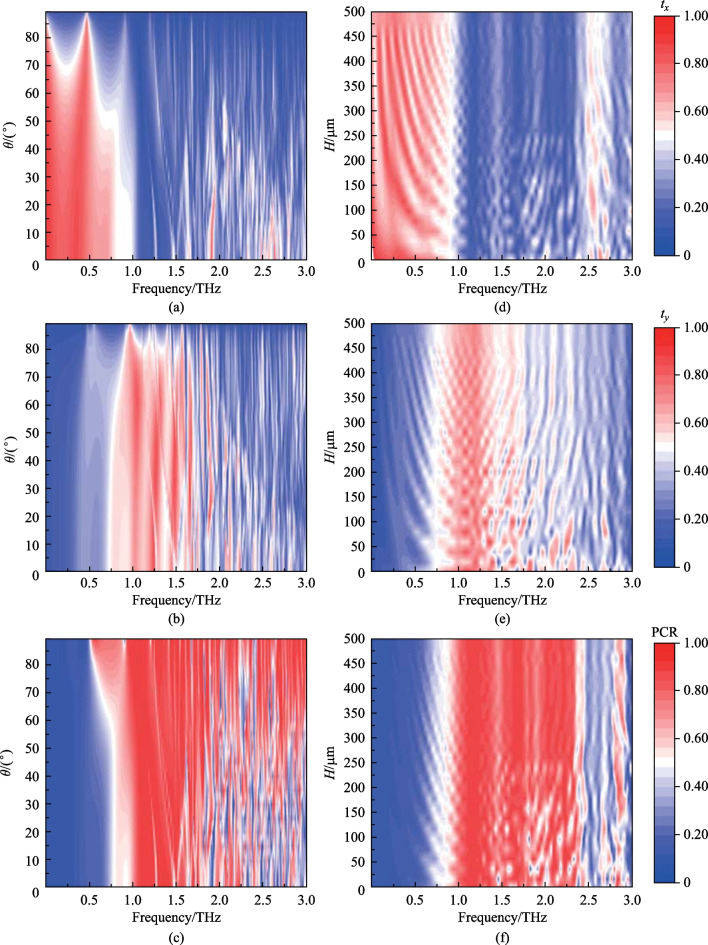
**a**–**c** Transmittance and PCR at different incident angles of the designed structure. Here, **a** and **b** represent the influence of different incident angles on the transmittance of terahertz waves in the *x*- and *y*-direction. **c** Represents the influence of different incident angles on PCR. **d**–**f** Transmittance and PCR at different substrate thicknesses. **d**, **e** represent transmittance of the *x*- and *y*-direction at different thicknesses. **f** represents PCR at different thicknesses. Here, the wider the red zone is, the better the device perform

When the two orthogonal components of the transmitted waves are equal, the linearly polarized waves can be converted into circularly polarized waves. The Stokes formula describing ellipticity, *χ* = *S*_1_/*S*_2_, where *S*_1_ and *S*_2_ are the Stokes parameters, is used to evaluate the circular polarization effect. Here, *S*_1_ = 2|*t*_*x*_||*t*_*y*_|sin*φ*, *S*_2_ =|*t*_*x*_|^2^ + |*t*_*y*_|^2^, and *φ* is the phase difference. From Fig. 4, when the *x*-polarized incident waves impinge on the device, the transmitted waves are completely converted into left-handed circularly polarized waves in 0.85 THz.

In Fig. 4, a* y*-polarized wave is incident into the device, then it will be converted into right-handed circularly polarized wave at 0.85 THz. Additionally, we use PB (Pancharatnam-Berry) phase [32, 33] to change the polarization state of transmitted waves. By adjusting the spatial transformation of this device, it can also achieve a relatively effective modulation effect.

**Fig. 5:**
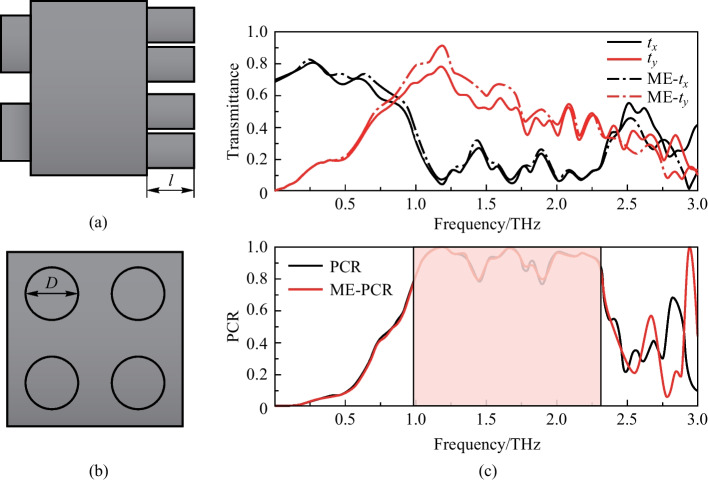
**a**, **b** Represents the microstructure with ME structure. *l*: height; *D*: diameter. **c** Influence of ME structure on transmittance and PCR, where solid line represents the device without ME structure, and dashed line represents adding ME structure. The red zone represents PCR exceeding 80%

We also consider the angular dependence of the proposed polarization convertor. (The substrate thickness is set to 10 μm here). The numerically resolved transmittance and PCR are depicted in Fig. 4. As shown in Fig. 4a, b, the transmittance decreases with the increase of incident angle. While the polarization conversion performance (PCR) is minimally affected (Fig. 5c). These results show that the structure has good robustness, which is of great significance for practical applications.

Regarding the application situation of the proposed convertor, the influence of substrate thickness on device performance is analyzed. As shown in Fig. 4d–f, the red ranges represent that the transmittance and PCR are better than white areas. The redder the area, the higher the transmittance and PCR. Thus, the device is more easily integrated by reducing the thickness of substrate.

### Optimization of polarization convertor

As shown in Fig. 5a–c, the incident angle seriously affects transmittance in *x*- and *y*-directions. With the angle increases, the transmittance decreases, which means that a lot of energy is lost in transmission. Therefore, it’s important to improve the energy efficiency of the device. We plate an anti-reflection film on the surface of the device to increase the transmittance [33]. Since the device adopts a metamaterial structure, the coating method is not applicable. A “moth-eye” (ME) structure is proposed to improve transmittance [34–36]. The ME structure is a periodic and regular arrangement of protrusions, as shown in Fig. 5a–b. It is always added at the bottom of the microstructure. Due to the protrusions on the surface, the reflected radiation is reduced, that is, the reflectivity is diminished and the transmittance is enhanced. Figure 5c shows the effect of the device on transmittance of terahertz waves after adding the ME structure. The ME structure with height *l* = 25 μm and diameter *D* = 35 μm have good influence on the performance of the device.

## Conclusion

We present a new all-silicon metamaterial featuring a cross-shaped microstructure capable of simultaneously achieving cross-linear and linear-to-circular polarization conversion in the terahertz frequency range. By analyzing the scanning results, we determine the optimal structural parameters. Our device achieves a broadband conversion range of approximately 1.32 THz and demonstrates high efficiency, reaching up to 80% for orthogonal linear polarization conversion. Furthermore, we investigate the device’s response at various incident angles and find that it exhibits no angular dependence. Additionally, we observe that the thickness of the substrate has minimal impact on the device’s performance, confirming its excellent integration potential. To further enhance the transmittance of terahertz waves, we incorporate a magneto-electric (ME) structure. Overall, our device serves as a valuable reference for the design and theoretical analysis of transmissive broadband polarization converters, particularly in integrated terahertz systems.

## Data Availability

The data that support the findings of this study are available from the corresponding author, upon reasonable request.
